# Resilience in the face of pelvic pain: A pilot study in males and females affected by urologic chronic pelvic pain

**DOI:** 10.1002/nau.24659

**Published:** 2021-03-25

**Authors:** Antonella Giannantoni, Marilena Gubbiotti, Matteo Balzarro, Emanuele Rubilotta

**Affiliations:** ^1^ Functional and Surgical Urology Unit, Department of Medical and Surgical Sciences and Neurosciences University of Siena Siena Italy; ^2^ Department of Urology San Donato Hospital Arezzo Italy; ^3^ Department of Urology A.O.U.I. Verona University Verona Italy

**Keywords:** catastrophizing, chronic pelvic pain, general distress, mood disorders, resilience

## Abstract

**Aims:**

Resilience represents a fundamental element in the experience of pain, as it allows adaptation to suffering and increases psychological social well‐being and quality of life (QoL). We investigated resilience in patients affected by urologic chronic pelvic pain (UCPP) and the relationships with pain severity and distribution, catastrophizing and psychological distress.

**Methods:**

Forty‐eight consecutive UCPP patients were classified on a pain body map as being affected by pelvic pain only or widespread pain (WP), and underwent the evaluation of resilience with the 14‐item Resilience Scale (RS‐14), with higher scores indicating high resilience levels; scores < 56 denote very poor resilience. Pelvic and nonpelvic pain intensity and the bother of urinary symptoms on QoL were measured by means of Pain Numerical Rating Scale (PNRS) and Visual Analog Scale (VAS). Pain Catastrophizing Scale (PCS) and Depression Anxiety Stress Scales (DASS‐21) investigated catastrophizing and psychological conditions.

**Results:**

Overall, RS‐14 mean ± *SD* total score was 50.2 ± 12.5 in patients with pelvic pain only and 40.2 ± 10.2 in those with WP. Significant relationships were observed between low resilience levels and high scores of pelvic and nonpelvic PNRS, VAS, pain catastrophizing scale and depression and anxiety, stress scale (for all: *p* < 0.001). Significantly lower RS‐14 scores were detected in females and in patients with WP.

**Conclusions:**

A very poor resilience has been identified in UCPP patients, particularly in those with greater catastrophizing and mood alterations. WP and female gender were mostly affected. In UCPP patients, low resilience appears as a crucial factor in pain experience.

## 1 INTRODUCTION

Interstitial cystitis (IC)/Bladder Pain Syndrome (BPS) is defined by the European Urological Association as persistent or recurrent pain perceived in the urinary bladder region, accompanied by pain worsening with bladder filling and/or day‐time and/or night‐time urinary frequency, in the absence of proven infection or other obvious local pathology.^1^ Patients affected by IC/BPS and by chronic prostatitis (CP)/chronic pelvic pain syndrome (CPPS) can present with chronic overlapping pain conditions (COPCs), in which pain affects both pelvic and nonpelvic regions of the body.^2^ Among COPCs, fibromyalgia, vulvodynia, temporomandibular disorder, chronic fatigue syndrome, irritable bowel syndrome (IBS), gastro‐oesophageal reflux disease, chronic migraine, are the most frequently reported.^3^ There is no unanimity on the cause of overlap; one hypothesis it represents the result of central sensitization with structural and functional changes in different central nervous system (CNS) areas, and long‐lasting, altered connectivity and plasticity in response to inflammation and neural injury.^4^ Whatever it is the causal mechanisms of COPCs, affected patients present with a marked vulnerability of psychosocial conditions.^2^, ^5^ In this regard, biopsychosocial models have explained the strong association between chronic pain, altered quality of life (QoL) and psychological factors like catastrophizing, with pain and psychological distress inducing negative effects on cognitive functioning and well‐being.^6^ Resilience allows patients to rebound from and to positively adapt to significant stressful events as in the case of chronic diseases.^7^ The concept of resilience varies according to the context in which it is used. In pain medicine resilience can be considered as the “capacity to adapt successfully to disturbances that threaten a patient's viability, function or development.”^8^ Resilience has a neurobiological substrate which involves both central and peripheral systems and processes underlying the stress response, such as the neuro‐endocrine pathway that plays a crucial role in adapting an organism to stressful events.^9^ Among the neural systems involved in resilience, the locus coeruleus/norepinephrine pathway, the mesolimbic reward circuit and the fear circuit play a fundamental role in controlling adaptive behaviors.^10^ Patients' resilience has been investigated in cancer and in non‐cancer chronic painful conditions, such as fibromyalgia, rheumatoid arthritis, systemic lupus erythematous, musculoskeletal pain,^11^ but currently, no information exist on resilience in urologic chronic pelvic pain (UCPP), and specifically in those presenting with COPCs. The aim of the present study was to investigate resilience in patients affected by UCPP and the relationships with pain severity and distribution, catastrophizing, and psychological distress.

## 2 PATIENTS AND METHODS

This pilot study involved 33 females and 15 males with UCPP who were regularly followed on an outpatient basis at three expert urology departments. Diagnosis of IC/BPS and CP/CPP was previously performed according to the European Society for the study of IC, with the exclusion of confusable diseases and to the Consensus Definition and Classification of Prostatitis from the National Institute of Health.^12^, ^13^ The study was conducted in respect to the Declaration of Helsinki and accepted by our clinical audit department (Institutional Review Board).

**Figure 1:**
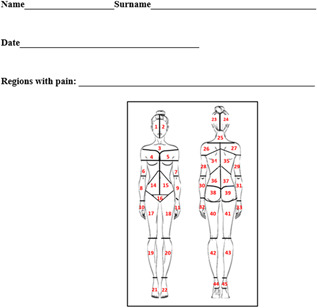
Pain body map (female)

**Figure 2:**
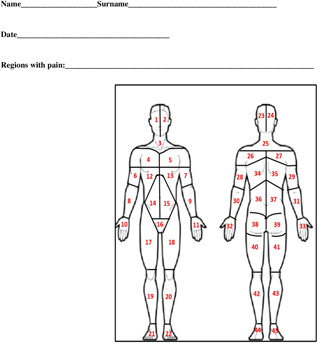
Pain body map (male)

Sociodemographic characteristics (age, education, marital status, and employment) and clinical data were collected in a clinical setting by in‐person interviews at the three out‐patient urology clinic departments. Collected information were then sent to our data management site for the final analysis. Patients were classified with a pain body map as being affected by pelvic pain only (PP only), pelvic pain and beyond the pelvis, and widespread pain (WP).^14^ Figures 1 and 2 show pain body map we use in our females and males with UCPP. The presence of COPCs was also assessed using the Complex Multiple Symptoms Inventory.^15^ Patients underwent the recording of the Pain Numerical Rating Scale (PNRS) to score both pelvic and nonpelvic pain intensity,^16^ and the Visual Analog Scale (VAS) to study the impact of urinary symptoms on QoL (0 = no bother and 10 = worst bother). Furthermore, they were asked to complete the following, self‐administered, questionnaires: Pain Catastrophizing Scale (PCS),^17^ Depression Anxiety and Stress Scale—short version (Depression Anxiety Stress Scales‐21 [DASS ‐21]) ^18^ and 14 item‐Resilience scale (RS‐14).^19^ Explanations on PNRS, VAS, PCS, and DASS‐21 have been previously described in detail.^20^ In brief, the 11‐point PNRS measured pelvic and nonpelvic pain intensity of our patients over the last 3 days.^16^ PCS is a 13‐item questionnaire assessing pain catastrophizing,^17^ which is described as the tendency to amplify the threat of a pain stimulus and to feel weak or helpless in the presence of pain, together with the inability to prevent or inhibit pain‐related thoughts, whether or not pain is present.^21^ Subjects who catastrophize tend to ruminate about their pain, to magnify their pain and to feel inadequate to manage their pain. Precisely, PCS includes three subscales which are rumination, magnification, and helplessness about pain. A 5‐point scale (from 0 =  not at all, to 4 = all the time) is used to calculate subscales scores, with a PCS total score ranging from 0 to 52. Higher measures denote greater report of catastrophizing cognition about pain experiences.^17^ The DASS‐21 investigates patients' general distress by considering three different psychological dimensions: anxiety (DASS‐21‐anxiety), depression (DASS‐21‐depression), and stress (DASS‐21‐stress). ^18^ Each item in the three dimensions is rated from 0 to 3 (0 = it never happened to me; 3 = it happened to me almost always). DASS‐21 total score ranges from 0 to 42, with higher scores representing more serious anxiety, depression, and stress.^18^

RS‐14 is a measure of resilience, with intrinsic properties highlighting individuals' positive psychological prerogatives rather than deficiencies, it includes a 7‐point rating (1 = strongly disagree; 7 = strongly agree) with scores ranging from 14 to 98.^19^ Scores < 56 denote very poor resilience, scores between 57 and 64 indicate low resilience, scores between 65 and 73 indicate resilience levels on the low end, scores between 74 and 81 indicate moderate resilience, scores between 82 and 90 a moderately high resilience and scores > 91 indicate high resilience levels. Five essential characteristics of resilience are represented in the RS‐14: “self‐reliance” (items 1, 5, 7, 12, and 14); “purpose” (items 2, 9, and 13); “equanimity” (items 3 and 10); “perseverance” (items 6 and 8) and “authenticity” (items 4 and 11).^19^

### 2.1 Statistical analysis

The Student *t* test and the Mann–Whitney *U* test were used to compare continuous parametric and nonparametric variables. *χ* ^2^ test with Yates' continuity correction or Fisher's exact test were used to test associations between categorical variables. The Pearson correlation assessed the relationships between variables. A two‐sided *p* < 0.05 was considered significant. All values are expressed as mean ± *SD*. Calculations were performed with IBM‐SPSS version 25.0 (IBM Corp).

## 3 RESULTS

From June to September 2020, 61 UCPP patients were prospectively observed and included in the study; 48 cases (33 females and 15 males) completed all the questionnaires and underwent the evaluation. All patients were under a multimodal treatment regimen based on the UPOINT system,^20^ including antimuscarinics, mirabegron, alpha‐blockers, antidepressants, anxiolytics, pregabalin, palmitoylethanolamide/polydatin, pelvic‐floor muscle exercises, applied in different combination modalities. Patients' demographics and characteristics of pain and COPCs are shown in Table 1. No significant difference was detected on mean ± *SD* age between patients with PP only and those with WP (46.8 ± 10.8 years and 47.9 ± 8.3 years, *p* = 0.8), while patients with WP had a trend to a significant longer disease duration as compared to those with PP only (7.0 ± 3.5 vs. 8.1 ± 4.2, *p* < 0.08).

**Table 1:** Sociodemographic, pain distribution, and chronic overlapping pain conditions in 48 urologic chronic pelvic pain patients

Variables	UCPP patients
Total no. of patients	48
Age (mean ± *SD*)	47.3 ± 9.5
Sex (males/females)	15/33
Disease duration (years; mean ± *SD*)	7.5 ± 3.8
Education (No. of patients, males/females)	
Compulsory	8 (3/3)
Further education	22 (7/15)
Higher education	18 (5/15)
Marital Status (No. of patients, males/females)	
Married	22 (6/13)
Single	13 (6/11)
Divorced	13 (3/9)
Working status (No. of patients, males/females)	
Worker	30 (10/20)
Jobless	16 (4/22)
Retired	2 (1/1)
Pain distribution (No. of patients, males/females)	
Pelvic pain only	12 (5/7)
WP pain	36 (10/26)
COPCs (No. of patients, males/females)	30 (9/21)
Chronic fatigue syndrome	11(3/8)
Endometriosis	6 (0/6)
Fibromyalgia	13 (0/13)
Gastroesophageal reflux	23 (9/14)
IBS	17 (8/9)
Low back pain	22 (8/14)
Osteoarthritis	2 (0/2)
Tension headache	9 (4/5)
TMD	8 (3/5)
Vulvodynia	4 (4/0)
Autoimmune diseases	5 (2/3)

Abbreviations: COPCs, chronic overlapping pain conditions; IBS, irritable bowel syndrome; TMD, temporo‐mandibular disorder; UCPP, urologic chronic pelvic pain.

On the pelvic pain site map, 10/48 patients (20.8%) presented with PP only, two with PP and beyond (4.1%) and 34/48 (70.9%) with WP (Table 2). Due to the small number, patients with PP and beyond were included in the same subgroup with WP patients. Associated COPCs were identified in 30/48 patients (62.5%): in most of cases, more than 1 COPC was observed (Table 1). The most frequently observed associations of COPCs were fibromyalgia, gastroesophageal reflux, and low back pain in females, and gastroesophageal reflux, IBS, and low back pain in males. Sjogren's syndrome had been diagnosed in three patients, lupus erithematosus systemic in two cases and scleroderma in one woman. Autoimmune diseases were found to be associated with vulvodynia, chronic fatigue syndrome, and fibromyalgia in women and with IBS and tension headache in males.

**Table 2:** Scores of pain numerical rating scale, visual analog scale (to measure the bother of urinary symptoms on quality of life), pain catastrophizing scale, depression, anxiety stress scale and 14‐item resilience scale in 48 patients affected by urologic chronic pelvic pain

Variables		UCPP patients with PP only (mean ± *SD*)			UCPP patients with WP (mean ± *SD*)	
	Total score	Males	Females	Total score	Males	Females
Pain Numerical Rating Scale	6.7 ± 0.4	7.1 ± 0.8*	6.3 ± 0.2	6.6 ± 0.5	6.5 ± 0.8	6.7 ± 0.3*§
Nonpelvic Pain Numerical Rating Scale	–	–	–	6.6 ± 1.1	6.4 ± 1.2	6.8 ± 1.3°°
ScaleS Score						
Visual Analog Scale	6.9 ± 0.2	6.8 ± 0.8	7.3 ± 0.7**	7.0 ± 1.3	6.2 ± 1.1	6.9 ± 1.2**
Pain Catastrophizing Scale	29.8 ± 9.7	27.3 ± 4.5	32.4 ± 5.1***	39.3 ± 12.6***	35.7 ± 4.2	42.9 ± 6.3***
21‐item Depression, Anxiety and Stress Scale	41.6 ± 3.9	41.5 ± 3.7	41.8 ± 4.1	51.3 ± 3.7§	49.6 ± 4.4	52.9 ± 5.6§°
14‐item Resilience Scale	50.2 ± 12.5§° 21.2 ± 5.9 19.0 ± 5.9	52.0 ± 13.6	48.5 ± 11.5§°	40.2 ± 10.2	41.9 ± 11.8	39.7 ± 8.7§§

Abbreviations: DASS‐21, 21‐item Depression Anxiety and Stress Scale; NP‐PNRS, Nonpelvic Pain Numerical Rating Scale; PCS, Pain Catastrophizing Scale; PNRS, Pain Numerical Rating Scale; UCPP, Urologic Chronic Pelvic Pain; VAS, Visual Analog Scale Score.

*p < 0.01 between males and females with PP only; *§*p* < 0.05 between females and males with WP.

**p < 0.001 between females and males with PP only, and between females and males with WP.

***p < 0.0001 between patients with WP and PP only, and between females and males with WP.

§*p* < 0.0001 between patients with WP and PP only; §§*p* < 0.01 between females and males with PP only; §°*p* < 0.01 between females and males with WP; °°*p* < 0.01 between males and females with WP.

### 3.1 Measures

The results of mean ± *SD* PNRS total scores are shown in Table 2. We did not find any significant difference on the mean ± *SD* pelvic PNRS total score between patients with PP only and those with WP. Among patients with PP only, pelvic PNRS scores were significantly higher in males as compared to females (*p* < 0.01). Among patients with WP, women presented with significantly higher scores as compared to men (Table 2). Overall, higher scores of nonpelvic PNRS (NP‐PNRS) were observed, which were significantly greater in women as compared to men (Table 2).

The results of VAS are showed in Table 2. We did not find significant differences on the mean ± *SD* VAS total score between the two subgroups of patients. Females were more bothered by their urinary symptoms than males, with a statistically significant difference among the two sexes in both the two subgroups of patients (Table 2).

When considering the results of PCS scale, we could observe high levels of catastrophizing in both subgroups of patients, but those with WP, and particularly females, were significantly more catastrophizing than patients with PP only (Table 2). PCS domains most affected in the two subgroups of patients are described in Table 3. Overall, the three PCS domains were more impaired in women, and particularly in those presenting with widespread pain. The results of DASS‐21 total and subscales scores are shown in Tables 2 and 4. Overall, patients with WP were significantly more affected by general distress as compared to those with PP only (Table 2). In PP only subgroup, males were affected by greater anxiety as compared to female; females with WP showed significantly higher levels of depression, anxiety, stress and general distress as compared to all patients with PP only (Table 4). With regard to RS‐14, the mean ± *SD* total score in patients with PP only was 50.2 ± 12.5, a value significantly higher than that measured in patients with WP (40.2 ± 10.2, *p* < 0.001) (Table 2). Overall, in both subgroups of UCPP patients very poor resilience levels were detected (Table 5).

**Table 3:** Pain Catastrophizing Scale: subscales scores in 48 urologic chronic pelvic pain patients

PCS subscale	PP only, males	PP only, females	WP, males	WP, females
	(mean ± *SD*)	(mean ± *SD*)	(mean ± *SD*)	(mean ± *SD*)
Helplessness	12.6 ± 5.5	16.2 ± 4.8*	16.5 ± 4.3	20.1 ± 6.9**
Magnification	6.6 ± 3.4	7.0 ± 5.1	8.3 ± 3.4*	10.4 ± 5.6**
Rumination	8.1 ± 4.7	9.2 ± 5.6	10.9 ± 5.0*	12.4 ± 6.3**
Total score	27.3 ± 4.5	32.4 ± 5.1	35.7 ± 4.2	42.9 ± 6.3***

Abbreviations: PCS, Pain Catastrophizing Scale; PP, pelvic pain Only; WP, widespread pain

* Between males with PP only and WP: *p* < 0.05.

** Between females with PP only and WP: *p* < 0.01.

*** Between males and females with WP: *p* < 0.001.

**Table 4:** 21 item Depression Anxiety and Stress Scale (DASS‐21) scores in 48 urologic pelvic pain patients

DASS‐21	PP only, males	PP only, females	WP, males	WP, females
Subscales and total scores	(Mean ± *SD*)	(Mean ± *SD*)	(Mean ± *SD*)	(Mean ± *SD*)
Depression score	13.2 ± 3.7	13.8 ± 4.1	18.3 ± 5.8*	19.8 ± 3.3*
Anxiety score	12.6 ± 3.9**	11.2 ± 4.5	13.4 ± 5.2	14.3 ± 4.7**
Stress score	15.7 ± 3.4	16.8 ± 3.7	19.5 ± 4.8***	18.4. ± 3.1***
Total score: general distress	41.5 ± 3.7	41.8 ± 4.1	49.6 ± 4.4	52.9 ± 5.6****

*Note*: Threshold values for depression, anxiety and stress case were set at ≥10, ≥8 and ≥15, respectively.

Abbreviations: DASS‐21, 21‐item Depression Anxiety and Stress Scale; PP, pelvic pain only; WP, widespread pain.

* Between males and females with WP and males and females with PP only: *p* < 0.001.

** Between males and females with PP only: *p* < 0.01; between females with WP and PP only: *p* < 0.001.

*** Between females and males with WP and females and males with PP only: *p* < 0.01.

**** Between WP and PP only total scores (females and males): *p* < 0.0001.

**Table 5:** Five essential characteristics of 14‐item Resilience Scale (RS‐14) in 48 urologic chronic pelvic pain patients

RS‐14 characteristics	UCPP patients with PP only, males (mean ± *SD*)	UCPP patients with PP only, females (mean ± *SD*)	UCPP patients with WP, males (mean ± *SD*)	UCPP patients with WP, females (mean ± *SD*)
Self‐reliance (items: 1, 5, 7, 12, 14)	6.2 ± 2.1*	5.9 ± 1.4	4.7 ± 1.3*	4.2 ± 0.9
Purpose (items: 2, 9, 13)	5.3 ± 2.2*	4.9 ± 1.8	4.1 ± 1.2*	3.8 ± 1.4
Equanimity (items: 3, 10)	5.2 ± 1.5*	4.8 ± 1.2	4.2 ± 1.1*	3.9 ± 1.0
Perseverance (items: 6, 8)	4.8 ± 1.2	4.6 ± 0.4	4.0 ± 1.1	3.8 ± 1.2
Authenticity (items: 4, 11)	5.0 ± 1.9*	4.7 ± 1.4	4.2 ± 1.2**	3.3 ± 1.4

Abbreviations: PP, pelvic pain; WP, widespread pain; UCPP, urologic chronic pelvic pain patients.

* *p* < 0.05 between males and females.

** *p* < 0.01 between males and females.

Overall, we could detect significant relationships between lower RS‐14 scores and higher pelvic and nonpelvic PNRS (*p* < 0.001 and *p* < 0.001, respectively). Table 6 shows correlation coefficients between RS‐14 and PNRS, VAS, PCS, and DASS‐21; all coefficients showed significant relationships between low scores of RS‐14 and high nonpelvic PNRS, VAS, PCS, and DASS‐21 scores. RS‐14 scores were significantly lower in women as compared to men (*p* < 0.000). RS‐14 items indicative of “authenticity” and “perseverance” were significantly lower in males and females with WP presenting with higher stress scores (*p* < 0.01 and *p* < 0.05, respectively). Other significant relationships were observed among RS‐14 and marital status and employment, with lower RS‐14 scores being detected in patients who were single and not engaged in work activity (*p* < 0.05 and *p* < 0.05, respectively). Finally, no significant relationship was detected between RS‐14 scores and duration of disease.

**Table 6:** Pearson correlation coefficients between 14‐item Resilience Scale and nonpelvic Pain Numerical Rating Scale, Visual Analog Scale, Pain Catastrophizing Scale, and Depression, Anxiety, and Stress Scale

Variable	NP‐PNRS	*p* level	VAS	*p* level	PCS	*p* level	DASS‐21	*p* level
RS‐14	−0.83	<0.001	−0.70	<0.001	−0.84	<0.001	−0.68	<0.001

Abbreviations: DASS‐21, 21‐item Depression, Anxiety, Stress Scale; NP‐PNRS, Nonpelvic Pain Numerical Rating Scale; PCS, Pain Catastrophizing Scale; RS‐14, 14 ‐item Resilience Scale; VAS, Visual Analog Scale.

## 4 DISCUSSION

The present study investigated resilience profile in a group of UCPP patients and assessed the relationships with pain intensity and distribution, catastrophizing, and general distress. We found very low resilience levels in UCPP patients, particularly in those presenting with WP and COPCs and, overall, in female gender. To our knowledge this the first study investigating resilience in patients with UCPP. In our study, patients with very poor resilience scores showed also high levels of catastrophizing, stress, and mood disorders.

While it is known that UCPP patients demonstrate catastrophizing beliefs about pain, especially when they show multiple comorbid conditions, and high rates of mood disorders and general distress,^22^ currently there are no consistent information about resilience in chronic pelvic pain. In a previous study conducted in patients with chronic painful conditions, aimed to translate and validate one scale used to measure resilience, unfortunately patients with pelvic pain were grouped with those presenting with low back pain, thus their resilience profile remained unclear.^23^ Although the present investigation is a pilot study performed in a limited number of patients, the results we observed were strengthened by measuring resilience in the context of other psychological factors such as catastrophizing, anxiety and depression using validated questionnaires. They represent the reason to perform a broader investigation on the field of resilience in UCPP patients.

Resilience is a multidimensional entity determined by the interplay among hereditary, biological, emotional, intellectual, and external factors and it ascribes to the process of adapting well in the face of adversity, and eventually return to preadversity status.^7^, ^8^ In pain medicine, resilience is now retained as an essential element in the experience of pain and in its treatment, as it alleviates suffering and increases psychosocial well‐being and QoL.^8^, ^9^ Previous observations in patients with different chronic pain conditions, such as low back pain, fibromyalgia, and rheumatoid arthritis, demonstrated that mood disorders and catastrophizing are important factors for both the emergence and evolution of the diseases.^24^ Currently, it is no clear whether catastrophizing is due to higher levels of pain and number of COPCs or vice‐versa. Several studies observed that maladaptive thoughts and feelings seem to play an important role in persistence of pain and that changes in catastrophizing are associated with improvement in pain intensity, depression levels, pain‐related anxiety and physical and psychosocial disabilities. ^25^ Reduced catastrophizing and emotional distress are instead identified in resilient individuals, who present with higher levels of pain acceptance.^7^, ^8^, ^9^

Indeed, resilience is not only a personality' trait but it involves specific neurobiological changes, such as modifications in the cerebral content of neurotransmitters,^9^ increased and prolonged blood levels of cortisol and glucocorticoids, increased levels of cytokines with consequent inflammation and atrophy in different areas of the CNS.^10^ In chronic pain states, altered plasticity and connectivity have been found in hippocampus, locus coeruleus/norepinephrine pathway, anterior cingulate cortex, prefrontal cortex, thalamus, cerebellum, central periaqueductal grey substance, mesolimbic reward circuit, and fear circuit.^26^ Not surprisingly, cerebral areas involved in pain processing are also engaged in mood regulation.^26^ These observations could explain, at least in part, the presence of long‐lasting and highly bothering urinary disturbances in many individuals with chronic pain and depression, as in patients in our study.

All our UCPP patients presented with a very poor resilience, which was particularly affected in cases with WP and multiple COPCs. There is no consensus on the causes of WP and COPCs, but it is generally retained they can be the result of central sensitization, with consequent, abnormal plasticity in several brain regions.^26^ The more consistent activation of both emotional and limbic cerebral regions in patients with persistent painful conditions could probably represent a determinant in the process of pain chronicization.^26^ It is possible to hypothesize that also in our UCPP patients, and particularly in those with WP, inefficient neural processing against the stressful event represented by their wider, chronic painful condition, may be the substrate of poor ability to adapt to pain, higher catastrophizing and distress accompanied by marked mood disorders. In this respect, it has been observed that, compared to individuals with normal or high resilience levels, low resilient subjects produce an exaggerated thalamus and insula activation, with abnormal emotional and vulnerability responses.^26^

Another notable finding was the gender difference in resilience we observed in the present study, with the three domains being more impaired in women and particularly in those with widespread pain. This is in contrast with the original validation study,^27^ in which no significant differences in resilience by sex or age were detected. Indeed, similarly to previous investigations, as compared to males, women reported greater pain catastrophizing and depression which could represent causative factors of reduced resilience.^7^, ^8^, ^9^ A similar negative emotionality has been described in a previous study on UCPP patients, although no investigation was conducted on patients' resilience profile.^28^ In recent times, many observations focused on pain catastrophizing and resilience as linked factors in pain adaptation processes.^29^ Thus, it is likely that if we control one of the two psychological conditions with appropriate treatments, the other will improve too. In this context, cognitive behavioral therapy (CBT) is a widely accepted treatment modality for chronic pain, with some investigations providing evidence on its ability in modifying cognitive content, such as catastrophic pain appraisals. Indeed, in a previous study conducted on CBT in few CP/CPPS patients, treatment significantly reduced patient disability, pain and catastrophizing within few treatment sessions.^21^ Similarly, improved psychological resilience has been observed to predict greater positive emotions, which in turn predict decreases in pain catastrophizing.^29^

The finding in our study of a poor resilience profile particularly in patients living alone and not engaged in work activity is in line with previous observations showing that emotional and social relationships improve resilience and well‐being.^9^, ^11^ Instead, no relationships were identified between resilience levels and duration of disease, probably due to the similar length of illness among our UCPP patients.

Resilience in our study was measured with the Italian version of RS‐14, which has been validated in a homogeneous group of young, unmarried females, with resultant scores appearing difficult to compare to those in the current study. Indeed, the validation study of the original RS‐14 was performed in middle aged and older adults.^27^ Subsequently, RS‐14 has been validated in several languages and used in differently aged subjects and in specific pathologic conditions,^30^, ^31^ and showed superior psychometric properties compared to other resilience scales.^31^ This made us confident in the use of the Italian version of RS‐14 in our UCPPS patients for the comparison of the results.

Limitations in our study were the small UCPP population included, and the higher number of females and cases with WP. Longitudinal data are needed to further elucidate resilience profile in UCPP patients, with deeper investigations about possible links between resilience, pain, and other psychological aspects. Worth of noting, all the patients we studied were under a phenotyping‐based, multimodal treatment strategy with beneficial effects on pain as compared to before treatment. Nevertheless, the results in our study induce us to state that we are still far from controlling all the pathological aspects of UCPP, specifically poor resilience, impaired mood, and catastrophizing beliefs, all making the experience of pain really a source of suffering, and requiring also resilience‐oriented interventions.

## 5 CONCLUSIONS

Patients affected by UCPP with greater catastrophizing, mood alterations, and severe general distress present with very low resilience levels. A common pathogenetic process could underpin the co‐occurrence of chronic pain, mood disorders, and low resilience.

## AUTHOR CONTRIBUTIONS

**Antonella Giannantoni**: conception and design of the study, data collection, drafting the article, critical revision of the article and final approval of the version to be published. **Marilena Gubbiotti**: data collection, data analysis and interpretation. **Matteo Balzarro**: data collection, data analysis and interpretation. **Emanuele Rubilotta**: data collection, critical revision of the manuscript, final approval of the version to be published.

## CONFLICT OF INTERESTS

The authors declare that there are no conflict of interests.

## Data Availability

Data available on request due to privacy/ethical restrictions.
